# The association between coaching behaviors and athlete burnout among college athletes: an integrated SEM–ANN model based on self-determination theory

**DOI:** 10.3389/fpsyg.2026.1802771

**Published:** 2026-06-26

**Authors:** Chen Chen, Hao Wu, Lei Zhang

**Affiliations:** 1 School of Physical Education, Henan University of Technology, Zhengzhou, Henan, China; 2 School of Kinesiology and Physical Education, Zhengzhou University, Zhengzhou, Henan, China

**Keywords:** autonomy, autonomy-supportive coaching, competence, controlling coaching, relatedness

## Abstract

**Background:**

As competitive training pressure continues to rise in higher education, athlete burnout among university students has become more prominent and is often accompanied by lower motivation, reduced performance, and stronger intentions to withdraw from sport. Although prior studies have examined the association between coaching behaviors and athlete burnout, less attention has focused on how this association relates to basic psychological needs, and few studies have combined theory-based testing with predictive analysis. Guided by Self-Determination Theory, this study tested a parallel mediation model including autonomy-supportive and controlling coaching behaviors, autonomy, competence, relatedness, and athlete burnout. The study also examined gender differences.

**Methods:**

Questionnaire data were collected from 800 university student athletes in China using standardized scales. A two-stage analysis combining partial least squares structural equation modeling and artificial neural network analysis was then conducted.

**Results:**

Autonomy-supportive coaching behaviors were negatively associated with athlete burnout, whereas controlling coaching behaviors were positively associated with burnout. Autonomy, competence, and relatedness were all negatively associated with burnout and partially accounted for the associations between coaching behaviors and burnout. Among these needs, competence showed a relatively stronger indirect association. Multi-group analysis further showed that the indirect association between autonomy-supportive coaching behaviors and athlete burnout through autonomy was clearer in female athletes.

**Conclusion:**

These findings offer empirical support for adjusting coaching communication, fostering need-supportive training environments, and developing targeted psychological support strategies in university athletic settings.

## 1 Introduction

Athlete burnout (AB) is a lasting negative mental state. It happens when athletes face long periods of training and competition pressure. AB mainly involves emotional and physical exhaustion, a lower sense of achievement, and less interest in sports (Liu and Li, 2024). In the last twenty years, the average rate of AB has been rising. Signs of AB appear in many ways. They include lower sport motivation, worse athletic performance, and higher stress. Burnout can also lead to leaving sports early (Cai et al., 2025). Studies on student athletes show that around 9.8% are at high risk of sport-related burnout (Saarinen et al., 2024).

AB does not have a single cause. It is likely connected to both external environments and internal mental processes. Coaching behaviors are an important part of the training context. It is closely related to the psychological experience of athletes (Zhang and Rhim, 2024). Research shows that different coaching styles can affect whether athletes’ basic psychological needs are met. These styles may also correspond to different patterns of burnout. For example, autonomy-supportive coaching (ASC) tends to be associated with stronger experiences of autonomy and competence. In contrast, controlling coaching (CC) more often co-occurs with need-thwarting experiences (Kim and Choi, 2024). On the other hand, internal psychological processes also matter for understanding burnout. Self-Determination Theory (SDT) proposes that when basic psychological needs for autonomy, competence, and relatedness are well satisfied, people usually show higher-quality motivation and better adjustment, and they often report lower burnout (Groenewal et al., 2021). Some studies also suggest that AB may have gender differences, with female athletes often reporting higher levels than male athletes (Saarinen et al., 2024). Many earlier studies used structural equation modeling (SEM) and latent profile analysis. They focused on linear links. They can test theory and estimate paths. They may miss nonlinear or complex patterns. The links among coaching behaviors, basic psychological needs, and burnout may be nonlinear. Artificial neural network (ANN) can add to this. A combined SEM–ANN approach supports theory testing and prediction. It also ranks variable importance.

In this study, we examined the link between coaching behaviors and AB at two levels. The external level covered coaching behaviors. The internal level focused on three SDT basic psychological needs. We used an SEM–ANN approach. This approach can test theoretical paths and identify key factors. It can also help describe predictive patterns.

SDT served as the theoretical framework for this study. The theory posits that autonomy, competence, and relatedness are basic psychological needs, and the extent to which these needs are satisfied is closely related to motivational states and psychological adjustment (Xu et al., 2025). In sports contexts, AB often manifests as a sustained decline in athlete engagement and a decrease in the quality of sport experience (Cai et al., 2025). From an SDT perspective, athletes who report limited choice during long-term training and competition, low confidence in their abilities, or weak social connections are more likely to report maladjustment and burnout (Çelik, 2025). Coaches’ guidance, communication, feedback, and team management are also closely associated with athletes’ experiences of autonomy, competence, and relatedness (Liu et al., 2025). Therefore, basic psychological need satisfaction was considered a theoretical mechanism for examining the associations between coaching behaviors and AB.

Many coaching behaviors show up in training and competition. These behaviors shape athletes’ daily experience. Many studies group coaches’ interpersonal styles into two types, ASC and CC. These two styles create different climates. They also relate to AB in different ways (Cho et al., 2019; Kim and Choi, 2024). ASC respects athletes’ choices and views. Coaches give reasons, provide informational feedback, and invite athletes into decisions. Athletes who perceive more ASC often report lower AB (Mossman et al., 2024). CC puts pressure on athletes and demands compliance. It can include threats, humiliation, conditional regard, and close monitoring. CC is linked to need frustration and higher AB (Choi, 2019; Morales-Sánchez et al., 2020). Therefore, H1 proposed that ASC would be negatively associated with AB, whereas H5 proposed that CC would be positively associated with AB.

Autonomy means a person can choose for themselves. A person can state what they want to do. A person can feel self-directed while acting (Ryan and Deci, 2000). In sport, athletes show autonomy when they feel respected. They also feel included in training plans, goal setting, and competition decisions. Research links autonomy to athletes’ motivation and psychological adjustment. When athletes feel more autonomy in training and competition, they often have stronger intrinsic motivation. They also report better sport experiences (Fan et al., 2023). Vilks and Kolesovs (2025) studied university athletes. They found higher autonomy went with lower AB. De Francisco et al. (2025) reported a similar pattern. Lower autonomy went with higher AB. Higher autonomy satisfaction often goes with less emotional exhaustion. It also goes with more stable sport engagement. Research shows that athletes who feel more supportive interactions from coaches often report higher autonomy satisfaction (Mossman et al., 2024). CC is linked to autonomy frustration (Morales-Sánchez et al., 2020). Some studies also examined autonomy and AB. Among college athletes, higher autonomy satisfaction often goes with lower AB. Athletes with higher autonomy usually had lower burnout scores. Many athletes with low autonomy had higher burnout scores (Vilks and Kolesovs, 2025). De Francisco et al. (2025) also found that autonomy satisfaction acted as a bridge between positive external factors and AB. This suggests that autonomy may be an important psychological pathway explaining how these variables connect. Based on this evidence, H2a proposed that ASC would be positively linked to autonomy; H3a proposed that autonomy would be negatively linked to AB; H4a proposed that autonomy would mediate the association between ASC and AB; H6a proposed that CC would be negatively linked to autonomy; and H7a proposed that autonomy would mediate the association between CC and AB.

Competence refers to a person’s feeling of being effective when doing tasks and reaching goals. In sport, competence is shown in how athletes see their ability and technical improvement. Conesa et al. (2020) found a clear negative link between competence satisfaction and AB. Athletes with higher competence often report lower burnout. De Francisco et al. (2020) found that athletes with lower competence often report higher burnout. Higher competence often comes with more stable sport engagement and less emotional exhaustion. Overall, higher competence often means lower exhaustion and more stable sport participation. Previous studies show that features of the social environment in sport are linked to athletes’ competence experiences. From the SDT view, ASC usually matches higher competence. For example, offering flexible options and giving constructive feedback often goes with a stronger sense of being capable (Cronin et al., 2022). CC, in contrast, is more likely to match competence frustration. Negative conditional regard and intimidation can lower athletes’ views of their ability. Competence frustration is a core part of this process (Morales-Sánchez et al., 2020). These research results show that different coaching styles may have different links with competence. The questionnaire study also shows a clear negative link between competence and AB. Athletes with higher competence often report lower burnout (De Francisco et al., 2020). Overall, coaching behaviors may affect AB in more than one way. They may also work through athletes’ sense of effectiveness in training. Based on this evidence, H2b proposed that ASC would be positively linked to competence; H3b proposed that competence would be negatively linked to AB; H4b proposed that competence would mediate the association between ASC and AB; H6b proposed that CC would be negatively linked to competence; and H7b proposed that competence would mediate the association between CC and AB.

Relatedness refers to feeling connected to others, accepted, and emotionally supported during activities (Vilks and Kolesovs, 2025). Conesa et al. (2020) found a clear negative link between relatedness and AB. Higher relatedness goes with lower overall AB and its dimensions. De Francisco et al. (2025) also found a strong negative link between relatedness and burnout in university athletes. When athletes get emotional support from teammates and feel cared for and understood by coaches, they report lower burnout. In more isolated training settings with little support, athletes often report lower relatedness and a higher burnout risk. Research evidence shows that ASC often goes with a stronger sense of team belonging and social support (Vilks and Kolesovs, 2025). CC often goes with social isolation and relatedness frustration (Mars et al., 2017). This suggests that coaching behaviors are linked to relatedness in different ways. Relatedness is closely linked to AB. A survey of university athletes found a clear negative link between relatedness and overall AB (De Francisco et al., 2025). This suggests that athletes’ social connection and support in training and competition are linked to their burnout level. Taken together, the interpersonal climate shaped by coaching behaviors may relate to AB through parallel changes in athletes’ relatedness experiences, which points to an indirect association pathway (Mossman et al., 2024). Based on this reasoning, H2c proposed that ASC would be positively associated with relatedness; H3c proposed that relatedness would be negatively associated with AB; H4c proposed that relatedness would mediate the association between ASC and AB; H6c proposed that CC would be negatively associated with relatedness; and H7c proposed that relatedness would mediate the association between CC and AB.

**Figure 1:**
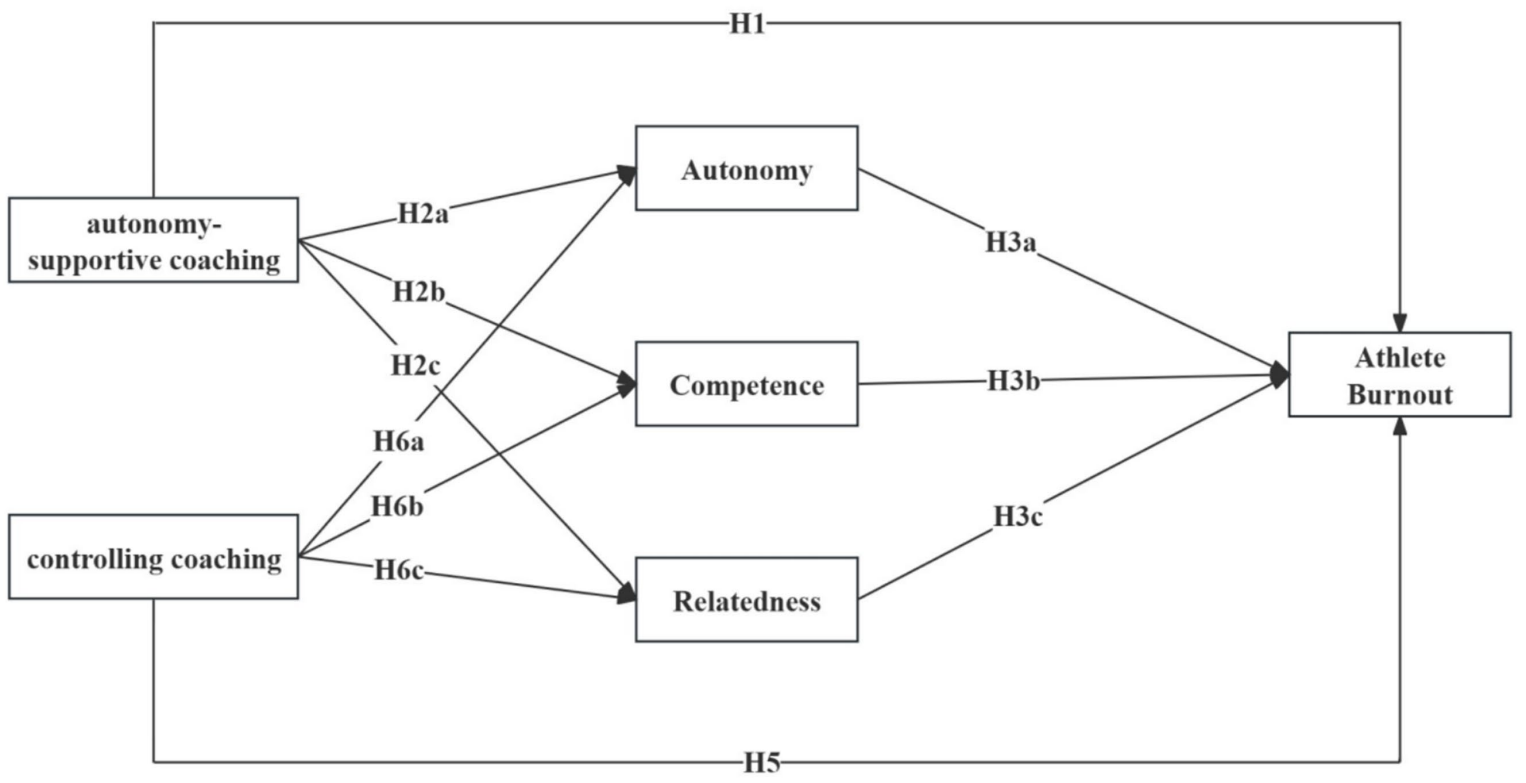
The hypothesized theoretical model.

Among college student athletes, AB has become a key concern that aligns closely with athletic performance and mental health. Prior work has provided initial evidence on how coaching behaviors and basic psychological needs relate to AB. Still, several gaps remain. First, research on coaching behaviors has examined ASC and CC in relation to AB, but fewer studies have compared these two coaching styles within the same theoretical framework. Studies also less often test whether ASC and CC show indirect associations with AB through basic psychological needs. Second, in SDT-based research, many studies have linked autonomy, competence, and relatedness to AB. However, researchers often focus on an overall index of basic psychological needs or on a single pathway. Fewer studies include all three needs in one model and compare their relative contributions as mediators. In addition, some work still explains AB mainly through a single factor. This approach may not fully capture how external training cues and internal psychological processes correspond within one integrated model. To address these gaps, this study used SDT as the guiding framework and examined autonomy, competence, and relatedness as parallel statistical mediators in the associations between ASC/CC and AB among college student athletes. We also compared the relative strength of these indirect associations. This design offers more detailed theoretical evidence for understanding the correlates of AB among college student athletes. The general objective was to examine how perceived coaching behaviors and basic psychological need satisfaction were associated with AB in a cross-sectional questionnaire design. The specific objectives were: (1) to examine the associations of ASC and CC with AB, corresponding to H1 and H5; (2) to examine the associations of ASC and CC with autonomy, competence, and relatedness, corresponding to H2a–H2c and H6a–H6c; (3) to examine the associations of autonomy, competence, and relatedness with AB, corresponding to H3a–H3c; and (4) to examine whether autonomy, competence, and relatedness showed indirect associations between ASC/CC and AB, corresponding to H4a–H4c and H7a–H7c. Based on the theoretical reasoning and prior literature, the hypothesized model is shown in Figure 1.

## 2 Materials and methods

### 2.1 Participants

We adopted a cross-sectional design. The participants were college student athletes majoring in physical education or sport-related programs in Chinese universities. To improve representativeness and reduce selection bias, we used random sampling in sport universities and universities with physical education programs in North, East, and South China (Guan et al., 2025). After university approval, we compiled a roster of eligible students at each participating university. We assigned a numeric code to each eligible student and conducted random selection separately within each university using a computer-generated random number table. This procedure reduced subjective selection and ensured that each eligible student on the roster had an equal probability of being invited to participate. During data collection, teachers explained the study, confidentiality, and voluntary participation. Students completed the questionnaires independently and anonymously. We ran the survey from November 2025 to January 2026. We used Sojump (www.Sojump.com) to send and collect the online questionnaires. We followed the 10:1 rule. Each item needed at least 10 respondents. The questionnaire included 61 items. We also allowed for an additional attrition rate of about 20%. Based on these criteria, the required sample size was set at 732 participants (Kline, 2023). All 876 distributed questionnaires were returned. We removed invalid questionnaires based on the following criteria: (1) more than 20% missing items, (2) selecting the same extreme option for over 80% of the items, and (3) an unusually short completion time, defined as less than one-third of the median (Wu et al., 2026). Following data screening, we retained 800 valid questionnaires. The effective response rate was 91.32%. Detailed sample characteristics appear in Table 1.

**Table 1:** Participant demographics (*N* = 800).

Participant profile	Category	Quantity	Percentage (%)
Gender	Male	395	49.4
	Female	405	50.6
Age	18–20	208	26.0
	20–22	174	21.8
	22–24	221	27.6
	> 24	197	24.6
Grade	Freshman	223	27.9
	Sophomore	180	22.5
	Junior	191	23.9
	Senior	206	25.8

### 2.2 Measures

#### 2.2.1 Autonomy-supportive coaching behaviors (autonomy-supportive coaching behaviors, SCQ)

We used the SCQ to assess athletes’ perceived level of ASC (Alcaraz et al., 2013). The scale includes six items. Participants responded on a 7-point Likert scale ranging from 1 (strongly disagree) to 7 (strongly agree). Higher scores indicate stronger perceived ASC. Prior research has validated the SCQ in Chinese athlete samples (Su et al., 2024). Confirmatory factor analysis (CFA) indicated acceptable model fit (χ2/df = 2.236, RMR = 0.025, GFI = 0.993, NFI = 0.995, IFI = 0.997, TLI = 0.995, CFI = 0.997, RMSEA = 0.039).

#### 2.2.2 Controlling coach behaviors (controlling coach behaviors scale, CCBS)

We used the CCBS to measure athletes’ perceived level of CC (Bartholomew et al., 2010). The scale includes 15 items and covers four dimensions: controlling use of rewards, negative conditional regard, intimidation, and excessive personal control. Participants rated each item on a 7-point Likert scale from 1 (strongly disagree) to 7 (strongly agree). Higher scores reflect more salient perceived CC. The CCBS has been validated among adolescent athletes (Bartholomew et al., 2010). CFA results supported the adequacy of the measurement model (χ2/df = 4.093, RMR = 0.044, GFI = 0.951, NFI = 0.973, IFI = 0.980, TLI = 0.970, CFI = 0.980, RMSEA = 0.062).

#### 2.2.3 Athlete burnout questionnaire (athlete burnout questionnaire, ABQ)

We used the ABQ to assess AB (Raedeke and Smith, 2001). The ABQ includes 15 items and captures three core dimensions of burnout: emotional/physical exhaustion, reduced sense of accomplishment, and sport devaluation. Participants responded on a 5-point Likert scale from 1 (almost never) to 5 (almost always). Items 1 and 14 were reverse scored. Higher scores indicate more severe burnout experiences. The ABQ has been validated among Chinese college student athletes majoring in physical education (Li R. et al., 2023). The CFA suggested a good fit (χ2/df = 2.151, RMR = 0.030, GFI = 0.974, NFI = 0.982, IFI = 0.990, TLI = 0.986, CFI = 0.990, RMSEA = 0.038).

#### 2.2.4 Basic psychological needs scale (basic psychological needs scale, BPNS)

We used the BPNS to measure the extent to which participants’ basic psychological needs are satisfied (Gagné, 2003). This instrument was developed within SDT and assesses three SDT core constructs: autonomy, competence, and relatedness. We used the Chinese version translated by Liu et al. (2013). The scale includes 19 items across three dimensions: autonomy, competence, and relatedness. Nine items are reverse scored. Participants rated the items on a 7-point scale from 1 (does not describe me at all) to 7 (describes me very well). Prior evidence supports the validity and reliability of the BPNS among Chinese college students (Du et al., 2023). The CFA showed an acceptable fit (χ2/df = 4.438, RMR = 0.048, GFI = 0.930, NFI = 0.931, IFI = 0.946, TLI = 0.929, CFI = 0.946, RMSEA = 0.066).

### 2.3 Data analysis

We used a two-stage strategy to test the theoretical model and check predictive performance. In Stage 1, we used partial least squares structural equation modeling (PLS-SEM) to assess the measurement model and the structural model. We estimated the path coefficients linking ASC and CC with AB. We also tested indirect effects through autonomy, competence, and relatedness (Leong et al., 2025). In Stage 2, we used ANN as a supplement. ANN supported prediction-based testing and showed variable importance. ANN is a “black box,” so we used its results for prediction, not for causal explanation (Almufarreh, 2024). The ANN model used ASC, CC, autonomy, competence, and relatedness as inputs. AB was the output. We trained and tested the model to obtain error indicators. We then used sensitivity analysis to calculate the relative and normalized importance of each input.

## 3 Results

### 3.1 SEM analysis

PLS-SEM was applied to assess the measurement and structural components. We selected PLS-SEM because the study aims to explain and predict the outcome variable, AB. In addition to testing direct paths and mediation effects, we emphasized indicators of variance explained and predictive utility, such as R^2^ and Q^2^, to evaluate predictive validity. This prediction oriented approach also fits well with models that include multiple mediators and multi group comparisons, because PLS-SEM readily supports bootstrapping for statistical inference (Hair et al., 2021).

### 3.2 Measurement model

For reflective measurement models, external loadings are often set at 0.708 or above. A loading of 0.60 or lower can still be acceptable when Composite Reliability (CR) and Average Variance Extracted (AVE) meet the standards and other items load well (Shela et al., 2023). We used a conservative deletion rule. We removed only items with loadings below 0.60. We did not delete items only because loadings were below 0.70. We checked CR, AVE, and content validity before decisions (Ketchen, 2013). We deleted one item from the relatedness scale due to an outer loading below 0.60. After removing this item, the CR and AVE values for relatedness reached acceptable levels. We also re-checked the item content and confirmed that the remaining items still reflected the core conceptual domain of relatedness; therefore, the deletion is unlikely to substantially reduce the content validity of this construct. We used AVE to assess convergent validity (Table 2). We used HTMT (Table 3) and the Fornell–Larcker criterion (Table 4) to assess discriminant validity. Overall, reliability and validity were acceptable.

**Table 2:** Reliability and validity results (*N* = 800).

Constructs	Items	Loadings	Cronbach’s α	CR	AVE
RE	RE1	0.620	0.853	0.869	0.585
	RE2	0.700			
	RE3	0.646			
	RE4	0.878			
	RE5	0.837			
	RE6	0.864			
CC	CC1	0.612	0.949	0.959	0.585
	CC2	0.654			
	CC3	0.662			
	CC4	0.709			
	CC5	0.773			
	CC6	0.777			
	CC7	0.804			
	CC8	0.804			
	CC9	0.827			
	CC10	0.818			
	CC11	0.841			
	CC12	0.834			
	CC13	0.710			
	CC14	0.807			
	CC15	0.788			
CO	CO1	0.683	0.826	0.831	0.539
	CO2	0.772			
	CO3	0.806			
	CO4	0.721			
	CO5	0.789			
	CO6	0.616			
AU	AU1	0.791	0.866	0.870	0.599
	AU2	0.807			
	AU3	0.700			
	AU4	0.810			
	AU5	0.787			
	AU6	0.744			
ASC	ASC1	0.771	0.926	0.928	0.631
	ASC2	0.864			
	ASC3	0.878			
	ASC4	0.877			
	ASC5	0.871			
	ASC6	0.861			
AB	AB1	0.780	0.945	0.948	0.565
	AB2	0.741			
	AB3	0.775			
	AB4	0.803			
	AB5	0.744			
	AB6	0.701			
	AB7	0.735			
	AB8	0.729			
	AB9	0.826			
	AB10	0.744			
	AB11	0.743			
	AB12	0.740			
	AB13	0.824			
	AB14	0.639			
	AB15	0.731			

ASC, autonomy-supportive coaching; CC, controlling coaching; AB, athlete burnout; AU, autonomy; CO, competence; RE, relatedness.

**Table 3:** Discriminant validity by HTMT (*N* = 800).

Constructs	RE	CC	CO	AU	ASC	AB
RE						
CC	0.321					
CO	0.435	0.486				
AU	0.352	0.276	0.560			
ASC	0.349	0.333	0.690	0.604		
AB	0.509	0.411	0.535	0.413	0.439	

ASC, autonomy-supportive coaching; CC, controlling coaching; AB, athlete burnout; AU, autonomy; CO, competence; RE, relatedness.

**Table 4:** Fornell–Larcker discriminant validity results (*N* = 800).

Constructs	RE	CC	CO	AU	ASC	AB
RE	**0.765**					
CC	−0.302	**0.765**				
CO	0.370	−0.449	**0.734**			
AU	0.311	−0.267	0.486	**0.774**		
ASC	0.313	−0.324	0.605	0.550	**0.855**	
AB	−0.466	0.409	−0.477	−0.382	−0.414	**0.752**

ASC, autonomy-supportive coaching; CC, controlling coaching; AB, athlete burnout; AU, autonomy; CO, competence; RE, relatedness. Bold italic values on the diagonal represent the square roots of the AVE for each construct.

### 3.3 Common method bias and confirmatory factor analysis

We used several methods to test common method bias. The Harman single-factor test showed that EFA extracted 10 factors with eigenvalues above 1. The first factor explained 30.806% of the variance. This was below the 40% threshold (Podsakoff and Organ, 1986). This suggests that common method bias is limited. We also added a common latent factor (CLF) to the measurement model. We compared it with the baseline model. The model difference was not significant (*p* > 0.05). The two results matched. We then ran CFA. Model fit was acceptable, with χ^2^/df = 3.237, SRMR = 0.052, GFI = 0.931, NFI = 0.922, IFI = 0.925, TLI = 0.918, CFI = 0.936, RMSEA = 0.042. According to Hu and Bentler (1998), the model fit was acceptable. Although these results suggest that common method bias was not a serious concern in the present data, the variables were measured using self-report questionnaires in a cross-sectional design. Therefore, potential common method bias cannot be completely ruled out.

### 3.4 Structural model

#### 3.4.1 Collinearity assessment

We used variance inflation factors (VIF) to check for possible collinearity (Hair et al., 2021). As shown in Table 5, all VIF values were below 3.3. This indicates that collinearity is unlikely to bias the model estimates.

**Table 5:** Assessment of multicollinearity in the structural model (*N* = 800).

Constructs	RE	CC	CO	AU	ASC	AB
RE						1.223
CC	1.118		1.118	1.118		1.291
CO						1.900
AU						1.540
ASC	1.118		1.118	1.118		1.836
AB						

ASC, autonomy-supportive coaching; CC, controlling coaching; AB, athlete burnout; AU, autonomy; CO, competence; RE, relatedness.

#### 3.4.2 Significance testing of structural paths

A 5,000-iteration resampling test was conducted to estimate path strength and statistical support. Following Hair et al. (2021), paths were considered significant when *t* exceeded 1.96, *p* was below 0.05, and the 95% interval estimates did not cross zero. All proposed paths satisfied these thresholds, supporting all hypotheses (Table 6). For AB, R^2^ was 0.373 and adjusted R^2^ was 0.369, indicating that the predictors accounted for 37.3% of its variance and that the model’s explanatory capacity remained stable. The Q^2^ value was 0.207, suggesting predictive relevance (Table 7).

**Table 6:** Structural model path significance (*N* = 800).

Path	Original estimate (O)	2.50%	97.50%	*t*	*p*	Results
ASC → RE	0.240	0.178	0.302	7.542	<0.001	Yes
ASC → CO	0.513	0.468	0.558	15.712	<0.001	Yes
ASC → AU	0.518	0.467	0.568	13.966	<0.001	Yes
ASC → AB	−0.102	−0.177	−0.026	2.629	0.009	Yes
CC → RE	−0.224	−0.288	−0.162	6.997	<0.001	Yes
CC → CO	−0.282	−0.334	−0.231	10.604	<0.001	Yes
CC → AU	−0.098	−0.159	−0.039	3.187	0.001	Yes
CC → AB	0.184	0.125	0.243	6.100	<0.001	Yes
RE → AB	−0.281	−0.341	−0.225	9.466	<0.001	Yes
CO → AB	−0.179	−0.252	−0.110	4.939	<0.001	Yes
AU → AB	−0.103	−0.170	−0.039	3.008	0.003	Yes

ASC, autonomy-supportive coaching; CC, controlling coaching; AB, athlete burnout; AU, autonomy; CO, competence; RE, relatedness.

**Table 7:** Model explanatory power (*N* = 800).

Constructs	R^2^	R^2^_adjusted_	Q^2^
AB	0.373	0.369	0.207

AB, athlete burnout.

#### 3.4.3 Mediation analysis

**Figure 2:**
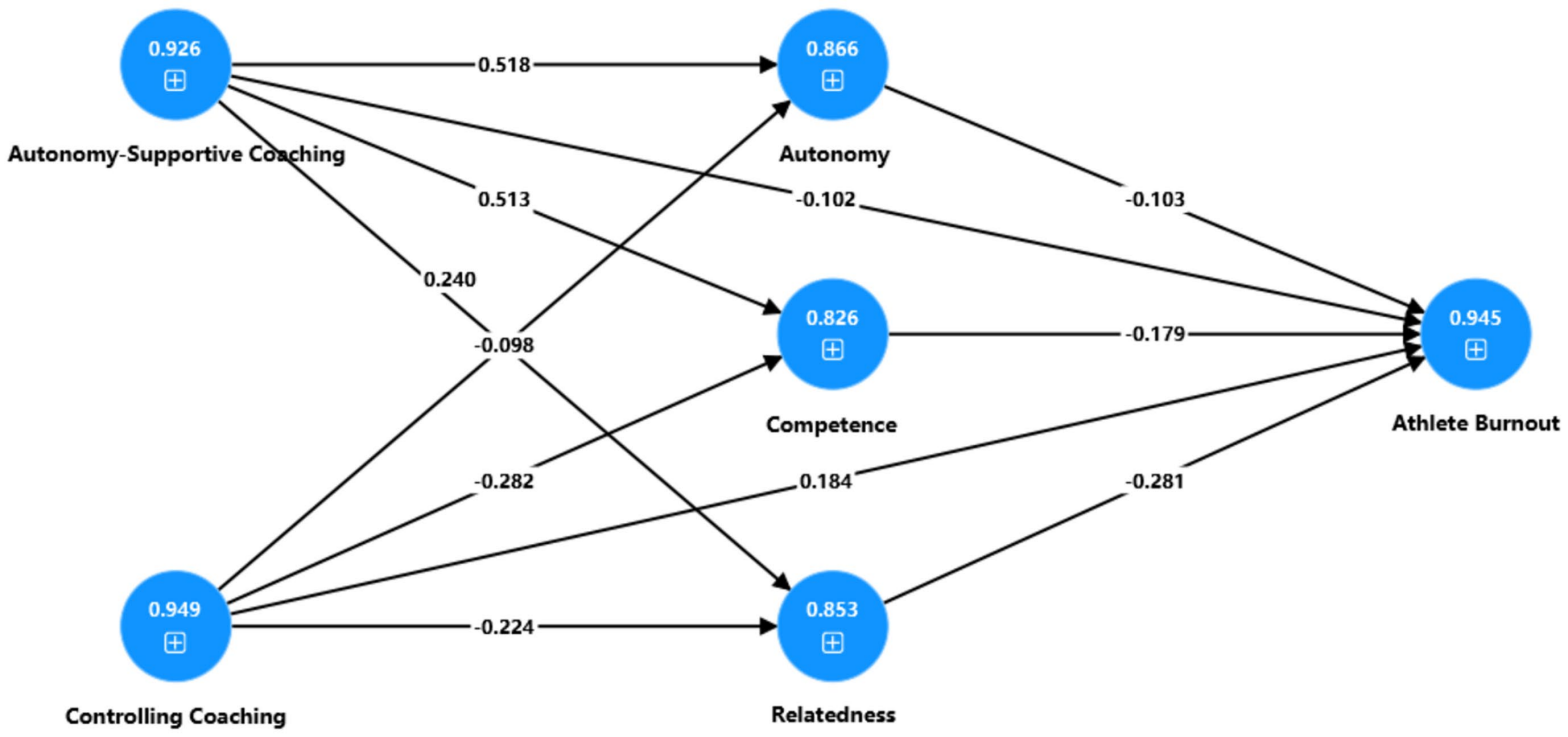
Mediation testing path diagram. Blue-circle values denote Cronbach’s alpha coefficients; path values denote path coefficients.

After controlling for covariates such as gender, age, and academic year, the results indicated that autonomy, competence, and relatedness each served as partial mediators in the associations between both types of coaching behaviors and AB. Table 8 presents the detailed mediation results. Figure 2 shows the overall path model with the mediation effects.

**Table 8:** Mediation effect results (*N* = 800).

Path	Indirect effect	2.50%	97.50%	*t*	*p*	Direct effect	2.50%	97.50%	*t*	*p*	Results
ASC → AU → AB	−0.053	−0.089	−0.020	2.975	0.003	−0.102	−0.177	−0.026	2.629	0.009	PM
ASC → CO → AB	−0.092	−0.131	−0.055	4.730	<0.001						PM
ASC → RE → AB	−0.067	−0.093	−0.046	5.605	<0.001						PM
CC → AU → AB	0.010	0.003	0.020	2.225	0.026	0.184	0.125	0.243	6.100	<0.001	PM
CC → CO → AB	0.050	0.031	0.074	4.582	<0.001						PM
CC → RE → AB	0.063	0.042	0.087	5.345	<0.001						PM

ASC, autonomy-supportive coaching; CC, controlling coaching; AB, athlete burnout; AU, autonomy; CO, competence; RE, relatedness; PM, partial mediation.

### 3.5 Gender-based path comparison

#### 3.5.1 MICOM-based invariance assessment

Measurement invariance across groups was examined using the measurement invariance of composite models (MICOM) procedure (Henseler et al., 2016). The results supported measurement invariance for all constructs (Table 9).

**Table 9:** MICOM Step 2_Compositional invariance: across Female vs. Male (*N* = 800).

Constructs	Correlation permutation mean	5% Quantile of empirical distribution of *c_u_*	*p*	Compositional invariance?
RE	0.999	0.996	0.626	Yes
CC	0.999	0.998	0.092	Yes
CO	0.999	0.998	0.480	Yes
AU	0.999	0.998	0.871	Yes
ASC	1.000	1.000	0.721	Yes
AB	1.000	0.999	0.642	Yes

ASC, autonomy-supportive coaching; CC, controlling coaching; AB, athlete burnout; AU, autonomy; CO, competence; RE, relatedness.

#### 3.5.2 Gender analysis

Group-based differences in path estimates were then tested. The ASC–autonomy–AB indirect association was stronger among female athletes (Table 10).

**Table 10:** Gender-based path estimates (*N* = 800).

Path	Original (Female)	Original (Male)	Difference (Female - Male)	2.50%	97.50%	*t*	*p*
ASC → AU → AB	−0.094	−0.020	−0.074	−0.153	−0.037	1.995	0.047

ASC, autonomy-supportive coaching; AB, athlete burnout; AU, autonomy.

### 3.6 Artificial neural networks

In the second-stage prediction analysis, we used the predictors that reached statistical significance in the PLS-SEM structural model as input neurons in the ANN. We used AB as the output. We operationalized AB as the mean score of the 5-point Likert items and treated it as a continuous variable. We introduced ANN to capture potential nonlinear relations and complex combined patterns without relying on normality assumptions. We also used it to provide an additional check on predictive performance (Teo et al., 2015). We built a multilayer perceptron model in the Neural Networks module of IBM SPSS and trained it using the backpropagation algorithm. We used the Sigmoid activation function for both the input-to-hidden and hidden-to-output layers (Sharma et al., 2019; Taneja and Arora, 2019). Consistent with Leong et al. (2018), the sample was split into training and testing subsets at an 80:20 ratio. The training error was 0.610 and the test error was 0.597. The two values were close, which suggests stable performance.

We used SPSS importance values for a sensitivity check. We normalized each value by dividing it by the largest one and reported percentages. Table 11 shows the ranking: relatedness (100%), competence (78.8%), CC (58.6%), autonomy (50.8%), ASC (11.1%).

**Table 11:** Normalized importance of each variable.

Constructs	Importance	Normalized importance
ASC	0.037	11.10%
CC	0.196	58.60%
AU	0.170	50.80%
CO	0.263	78.80%
RE	0.334	100.00%

ASC, autonomy-supportive coaching; CC, controlling coaching; AU, autonomy; CO, competence; RE, relatedness.

## 4 Discussion

This study found that ASC was negatively correlated with AB. CC was positively correlated with AB. These results support Hypotheses H1 and H5. The findings show that different coaching styles match different burnout patterns. ASC is linked to lower AB. This matches Mossman et al. (2024). When coaches give athletes more choices and listen to their views, athletes often report less emotional exhaustion and less sport devaluation. From the SDT view, these interaction styles often go with higher autonomy satisfaction and stronger behavioral engagement. This pattern fits lower burnout levels (Carroll and Allen, 2021). CC is linked to higher AB. This matches Morales-Sánchez et al. (2020) and Choi (2019). Coaching styles with threats, humiliation, or conditional regard often go with more psychological need frustration and higher burnout. In Chinese sports training, discipline and performance have been emphasized. This can make CC seem culturally acceptable to some extent (Zhu et al., 2024). However, the findings show that too much control still leads to worse psychological experiences for athletes. This suggests that moving toward more autonomy-supportive coaching may help reduce AB in training.

This study also found that ASC was significantly positively associated with autonomy, whereas CC was significantly negatively associated with autonomy. Autonomy was also significantly negatively associated with AB. Mediation results further showed that autonomy partially mediated the associations between ASC and AB and between CC and AB. These findings support Hypotheses H2a, H3a, H4a, H6a, and H7a. Overall, autonomy appears to be a key psychological variable for understanding how coaching behaviors relate to AB. More specifically, when athletes perceive that coaches provide choice, explain the purpose of training tasks, and respect athletes’ viewpoints, athletes tend to report higher autonomy. This experience often co-occurs with lower burnout (Mossman et al., 2024). In contrast, CC tends to align with lower autonomy. It may also coincide with stronger perceived external pressure and higher burnout experiences (Morales-Sánchez et al., 2020). The negative association between autonomy and burnout in our study also matches prior findings. Vilks and Kolesovs (2025) reported that athletes with higher autonomy typically report lower emotional exhaustion and less sport devaluation. Taken together, the present findings show a stable indirect association pattern in which autonomy links both types of coaching behaviors with AB. This pattern matches the evidence summarized by De Francisco et al. (2025). Their review also pointed to autonomy as an indirect pathway between external factors and burnout. In the Chinese sport context, traditional training models often stress authority and compliance. Even in this context, our results show that autonomy-supportive tendencies link with higher autonomy experiences. These tendencies also link with lower burnout experiences. This finding can guide practice adjustments in similar settings (Li Y. F. et al., 2023).

The results show that ASC is positively correlated with competence. CC is negatively correlated with competence. Competence is also negatively correlated with AB. The mediation analysis indicates that competence plays a partial mediating role between ASC and AB. It also plays a partial mediating role between CC and AB. These results support Hypotheses H2b, H3b, H4b, H6b, and H7b. The positive link between ASC and competence matches previous research. Mossman et al. (2024) found that when coaches provide clear feedback, set appropriate challenging goals, and recognize athletes’ efforts, athletes feel more competent during training. The negative link between CC and competence matches (Morales-Sánchez et al., 2020). CC often involves excessive criticism or a focus only on results. These behaviors can lower athletes’ views of their ability and increase their sense of failure. The results also support the negative link between competence and AB. When athletes feel little progress or think they cannot improve, they report more emotional exhaustion and more sport devaluation (De Francisco et al., 2025). From the SDT view, competence is an important psychological pathway linking coaching behaviors and burnout. This matches earlier findings (Cronin et al., 2022; Morales-Sánchez et al., 2020; Vilks and Kolesovs, 2025).

Our results showed a positive link between ASC and relatedness. CC showed a negative link with relatedness. Relatedness showed a negative link with AB. Mediation tests showed that relatedness partly explained the links from ASC to AB. It also partly explained the links from CC to AB. These results support H2c, H3c, H4c, H6c, and H7c. ASC can raise relatedness. Coaches who show respect, understanding, and emotional support help athletes feel accepted and valued (Cronin et al., 2022). This helps athletes build stable connections. CC can lower relatedness. Humiliation, neglect, and conditional regard can weaken athletes’ emotional ties with coaches and teammates (Mars et al., 2017). These experiences can increase social disconnection. We also found that higher relatedness went with lower AB. This matches (De Francisco et al., 2025). When athletes lack stable interpersonal support, they often report more emotional exhaustion and more sport devaluation. In SDT terms, ASC may relate to lower burnout through stronger social connection. CC may link to higher burnout in part through weaker social connection. In the mediation model, relatedness acted as a bridge from both coaching behavior types to AB. Coaching behaviors may relate to burnout through athletes’ own views of relationship quality and social support. These views then link with burnout experiences.

In the mediation analysis, the strongest indirect pathway ran from ASC to competence and then to AB. The weakest pathway ran from CC to autonomy and then to AB. This pattern shows that coaching behaviors link to AB through different psychological processes. These indirect links also differ in strength. The indirect link from ASC to AB through competence was the strongest. One possible reason is that ASC may match athletes’ internal need satisfaction well. Coaches may encourage athletes to share their views. Coaches may also give room for choice. Coaches may also give respectful feedback. In this setting, athletes often report stronger competence (Cronin et al., 2022). Athletes may feel more capable. Athletes may also feel more confident. These positive experiences often go with lower burnout experiences (Conesa et al., 2020). Because competence is closely linked to how athletes evaluate their ability and maintain training engagement, it showed a comparatively strong mediating role in our model. By contrast, the indirect pathway from CC to autonomy and then to AB was relatively weak. Although CC may coincide with lower autonomy through external pressure and excessive monitoring, the link between reduced autonomy and AB appeared less pronounced in this specific pathway. One possible explanation is that the correlates of CC may not center only on autonomy. CC may also align with AB through other processes, such as stronger anxiety and stress and weaker perceived social support (De Francisco et al., 2025). In other words, although lower autonomy relates to burnout, this association was weaker here than the indirect pattern linked to ASC and the positive competence-related experience.

In addition, we used ANN to compare the relative importance of significant predictors of AB from a predictive perspective. The results showed that relatedness had the highest importance among all input variables, followed by competence. In contrast, ASC and CC carried lower relative weights in the multivariable prediction. This pattern does not imply that relatedness is necessarily stronger than other variables at a causal level. Instead, it suggests that within a combined prediction setting, athletes’ experiences of social connection and belonging occupy a more central place in predicting AB. This finding fits the SDT focus on basic psychological needs. It also shows that, in practice, programs may gain benefits from stronger peer relationships and a better team climate. Programs also need to pay attention to coaching style.

We also point out that mediation results need two separate readings. One reading is statistical significance. The other reading is effect size. Some indirect effects were small in absolute value. Their confidence intervals still did not include zero. This result shows a clear and steady statistical difference in these indirect pathways. At the same time, a small effect does not mean the pathway has no practical value. It can mean coaching behaviors link to burnout through several psychological processes. These processes work at the same time. This view also gives practical points for intervention. A more suitable reading of our results is this. Basic psychological needs show indirect associations between coaching behaviors and AB. These indirect associations are present. Their size is modest. In the full predictive pattern, relatedness and competence had higher importance. This result suggests that interventions may focus more on team connection and competence experiences. Interventions may also improve coaching behaviors. These parts can work together. They can act as layered protective factors.

The indirect path from ASC to autonomy and then to AB differed significantly by gender, with a stronger indirect association in female athletes. This pattern suggests that the autonomy-related pathway may be relatively more salient for understanding reported AB patterns among female athletes in the present sample. This interpretation is broadly consistent with prior evidence suggesting that female athletes may be more responsive to supportive coaching interactions and interpersonal dynamics in the coach-athlete context (Kim and Cruz, 2022). In the present model, higher autonomy was associated with lower AB. From this perspective, respect, acknowledgment, and understanding conveyed through ASC may be more likely to align with female athletes’ autonomy-related experiences, which may help explain the stronger indirect association observed in the female group (Cronin et al., 2019; Poucher et al., 2018). The indirect path was weaker in male athletes. This does not indicate that autonomy is unimportant for male athletes. Rather, in the present model, it may suggest that the association between coaching behaviors and AB in male athletes is distributed across a broader combination of psychological needs and situational factors. More broadly, gendered patterns in sport-related psychological processes may also shape how athletes respond to coaching contexts (Ojio et al., 2025). These results highlight the potential value of gender-sensitive approaches. For female athletes, autonomy-supportive strategies may be especially relevant to their reported autonomy and AB patterns. For male athletes, support strategies may benefit from considering a wider set of training and contextual factors, rather than emphasizing a single pathway alone.

## 5 Implications

### 5.1 Theoretical implications

First, many earlier studies mainly used linear models. We used a two-stage approach with PLS-SEM and ANN. This is a methodological contribution. PLS is a linear and compensatory model. One predictor can rise when another drops. This may not hold for AB. Positive coaching behaviors may not make up for stress or negative behaviors. ANN is nonlinear and non-compensatory. It can capture complex patterns that linear models miss. ANN can also rank the most important predictors. It reports prediction accuracy. This adds support for theory testing.

Second, within the SDT framework, our results show how external training cues, like coaching behaviors, relate to athletes’ experiences of autonomy, competence, and relatedness. These need experiences are systematically linked to emotional exhaustion and burnout. By putting contextual factors and internal psychological processes in one chain and testing the mediating roles of the three needs, this study reinforces the situational need-outcome logic. It extends SDT to competitive sport and high-pressure training, providing a context-sensitive way to understand burnout.

Third, we compared autonomy, competence, and relatedness as mediators between coaching behaviors and AB. We tested each indirect path and identified the strongest one. The strongest path ran from ASC to competence to AB. This suggests that competence may be a key link between coaching support and lower burnout. This finding also helps refine the theoretical interpretation of need-based mechanisms by indicating that autonomy, competence, and relatedness may not operate with equal salience in the coaching-burnout association. In addition, the observed gender difference suggests that, although competence was the strongest overall mediator, autonomy-related pathways may be relatively more salient in some subgroups (particularly female athletes), which adds nuance to the interpretation of coaching mechanisms in sport settings. This result can guide future studies on mechanisms, subgroup tests, and longitudinal work. It also suggests practical focus. Coaches can build competence through feedback, goal setting, and skill mastery in training.

### 5.2 Practical implications

First, coaches should use more ASC and limit over-control. Coaches can ask for athletes’ views. Coaches can invite athletes into decisions. Coaches can give clear and meaningful feedback. These actions support autonomy and competence. They often go with lower AB. They can also help athletes keep motivation and feel less exhausted in training.

Second, athletes can look for more autonomy in daily training. Athletes can talk with coaches in a calm and direct way. Athletes can join training decisions and state personal goals. These actions can raise a sense of effectiveness and help keep motivation. Athletes can also build team support. Athletes can ask teammates and coaches for help in competition. These steps may lower burnout and support well-being.

Third, the observed gender difference suggests that practical support in college sport may need to be more gender-sensitive. In the present findings, female athletes showed a more salient autonomy-related pathway, so coaches may place greater emphasis on autonomy-supportive communication, such as offering meaningful choices, acknowledging athletes’ perspectives, and providing emotional support. For male athletes, coaches may also consider combining autonomy support with skill challenge and task-focused feedback to improve the fit of support strategies. A gender-sensitive approach may better support athletes’ adjustment and address burnout-related experiences in college sport.

## 6 Study limitations and future research

First, this study used a cross-sectional design. It cannot show change over time. It also cannot support strong causal claims. Future work can use longitudinal designs or experiments. Second, the data were mainly self-report, which may introduce social desirability bias and potential common method bias. Although the Harman single-factor test and the common latent factor comparison suggested that common method bias was limited, this possibility cannot be fully excluded in a cross-sectional questionnaire design. Future work can use multi-source data (e.g., coach ratings, peer reports), time-lagged designs, observations, daily logs, or experience sampling to further reduce method-related bias. Third, we did not separate sport types. Sport type may change training pressure and coach–athlete interaction. Future work can compare individual and team sports. Finally, the sample was Chinese college athletes. Culture and age stage may limit generalization. Future work can test the model in other countries and age groups.

## 7 Conclusion

Guided by SDT, this cross-sectional questionnaire study surveyed 800 Chinese college student athletes and used an integrated PLS-SEM and ANN analytic strategy to examine the associations among ASC, CC, basic psychological needs, and AB. The findings indicated that ASC and CC corresponded to different burnout-related patterns, with ASC aligning with lower reported AB and CC aligning with higher reported AB. Autonomy, competence, and relatedness were also meaningfully associated with both coaching behaviors and AB, suggesting that basic psychological need satisfaction is an important psychological context for understanding athletes’ burnout-related experiences. The parallel mediation findings further showed that autonomy, competence, and relatedness formed indirect association pathways between coaching behaviors and AB, with competence showing a comparatively stronger indirect association in the overall model. The gender analysis suggested that the autonomy-related indirect pathway between ASC and AB was more salient among female athletes. In addition, the ANN analysis identified relatedness and competence as the most prominent variables for AB prediction within the multivariable model. Taken together, these findings summarize the association patterns among coaching behaviors, basic psychological needs, and AB, and may offer empirical reference points for need-oriented training management and psychological support in collegiate sport settings.

## Data Availability

The datasets presented in this study can be found in online repositories. The names of the repository/repositories and accession number(s) can be found in the article/supplementary material.
